# Toward spatio‐temporal delineation of positive interactions in ecology

**DOI:** 10.1002/ece3.6616

**Published:** 2020-08-06

**Authors:** Benjamin B. Tumolo, Leonardo Calle, Heidi E. Anderson, Michelle A. Briggs, Sam Carlson, Michael J. MacDonald, J. Holden Reinert, Lindsey K. Albertson

**Affiliations:** ^1^ Department of Ecology Montana State University Bozeman MT USA; ^2^ Department of Forest Management W.A. Franke College of Forestry and Conservation University of Montana Bozeman MT USA; ^3^ Department of Land Resources and Environmental Sciences Montana State University Bozeman MT USA

**Keywords:** biodiversity, ecosystem engineering, facilitation, organism interaction, scaling, traits

## Abstract

Given unprecedented rates of biodiversity loss, there is an urgency to better understand the ecological consequences of interactions among organisms that may lost or altered. Positive interactions among organisms of the same or different species that directly or indirectly improve performance of at least one participant can structure populations and communities and control ecosystem process. However, we are still in need of synthetic approaches to better understand how positive interactions scale spatio‐temporally across a range of taxa and ecosystems. Here, we synthesize two complementary approaches to more rigorously describe positive interactions and their consequences among organisms, across taxa, and over spatio‐temporal scales. In the first approach, which we call the mechanistic approach, we make a distinction between two principal mechanisms of facilitation—habitat modification and resource modification. Considering the differences in these two mechanisms is critical because it delineates the potential spatio‐temporal bounds over which a positive interaction can occur. We offer guidance on improved sampling regimes for quantification of these mechanistic interactions and their consequences. Second, we present a trait‐based approach in which traits of facilitators or traits of beneficiaries can modulate their magnitude of effect or how they respond to either of the positive interaction mechanisms, respectively. Therefore, both approaches can be integrated together by quantifying the degree to which a focal facilitator's or beneficiary's traits explain the magnitude of a positive effect in space and time. Furthermore, we demonstrate how field measurements and analytical techniques can be used to collect and analyze data to test the predictions presented herein. We conclude by discussing how these approaches can be applied to contemporary challenges in ecology, such as conservation and restoration and suggest avenues for future research.

## Graphical abstract

Given current and predicted future levels of global change and biodiversity loss, there is an urgent need to better understand the consequences of organism interactions that might be lost or altered. This urgency is especially pertinent for positive interactions (facilitation) that influence an organism's environmental tolerance and expand its niche space. In this paper, we address the improved integration of spatio‐temporal dynamics into positive interactions across taxa and ecosystems with two approaches, accompanying hypotheses and analytical techniques.

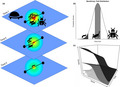

## 1 INTRODUCTION

Given current and predicted future levels of global change and biodiversity loss, there is an urgent need to better understand organism interactions that might be lost or altered (Bulleri, Bruno, Silliman, & Stachowicz, 2016; HilleRisLambers, Harsch, Ettinger, Ford, & Theobald, 2013; Kéfi et al., 2012; Wright, Wardle, Callaway, & Gaxiola, 2017). This urgency is especially pertinent for positive interactions, defined as interactions between organisms of the same or different species that directly or indirectly improve performance of at least one of the participants (Table 1; Bertness & Callaway, 1994; Bronstein, 1994). Understanding how positive interactions will be lost or altered in the context of global change is important as these interactions are known to increase organismal fitness and population abundance, maintain species coexistence within communities, and alter ecosystem functions such as biogeochemical cycling (Bulleri et al., 2016; Gross, 2008; Wright et al., 2017). Despite the appreciated importance of positive interactions to ecosystem function, a general understanding of how positive interactions operate and change across taxa and over spatio‐temporal scales remains an active area of research (Calatayud et al., 2019; Lam & Chisholm, 2020; Silknetter et al., 2020).

**TABLE 1:** Glossary of terms used in this article

Term	Definition
Amelioration	Environmental improvement resulting in a reduction in stress experienced by an individual; often organism‐mediated through habitat or resource modification
Beneficiary	An organism that receives positive effects from the presence or actions of another organism. It is the facilitatee
Ecosystem engineer	An organism that modulates habitat and resource availability (other than themselves) by modifying physical environmental conditions.
Facilitation	Organism‐mediated positive effects resulting in improved performance of at least one participant in the interaction
Facilitator	An organism that provides positive effects to another organism by modulating habitat and resource availability (other than themselves) through modification of physical environmental conditions
Performance	A functional metric directly related to an individual's ability to survive and reproduce, often measured as presence, density, biomass, or body growth
Positive interaction	Interactions between organisms of the same or different species that directly or indirectly improve performance of at least one
Stress	The downregulation of organismal performance through environmental or biological interaction
Traits	Attributes of individuals that inform ecological function and role such as: ontogeny, body size, mobility, and trophic position

In this paper, we begin by summarizing the recognized significance of spatio‐temporal dynamics in modulating the importance of positive interactions to organism performance and ecological processes. Following this introduction, we synthesize two complementary approaches and accompanying hypotheses for improved measurement of positive interactions over space and time that can be applied across a range of taxa and ecosystems. In approach 1, we present hypotheses on the explicit spatio‐temporal extent of positive interactions based on two principal mechanisms: habitat modification and resource modification. In approach 2, we present hypotheses for the ways that traits and trait variation of facilitators and beneficiaries may change the strength of positive interactions over space and time (Table 1). We then demonstrate how these two approaches can be integrated together and how measurements can be taken for analysis of the spatio‐temporal dynamics of positive interactions. We conclude by discussing how these approaches can be applied to contemporary ecological challenges such as restoring degraded habitats and by providing suggestions for future research directions.

## 2 SPATIO‐TEMPORAL SCALE IN POSITIVE INTERACTION

Spatio‐temporal scaling has been central to the study of ecology for decades (Gonzalez et al., 2020; O'Neill, Deangelis, Waide, Allen, & Allen, 1986). Sub‐disciplines concerned with organism interactions are no exception. Within the positive interaction literature, a growing body of work shows the importance of spatio‐temporal scale in changing the strength of positive interactions ranging from interspecific facilitations to interkingdom mutualisms (Table 1; Bertness & Callaway, 1994; Bronstein, 1994; Bruno, Stachowicz, & Bertness, 2003). The most foundational framework of ecological positive interactions in space and time is arguably the stress gradient hypothesis (SGH), which predicts that organisms in more stressful environments experience a higher frequency of positive interactions (Bertness & Callaway, 1994). Since its formalization, there has been support for both the simplicity and utility of the SGH (Bruno et al., 2003; Callaway et al., 2002; He, Bertness, & Altieri, 2013), however see counterexamples in extremely stressful environments (He & Bertness, 2014; Maestre, Callaway, Valladares, & Lortie, 2009). Despite the progress that has been made by directly testing the SGH, literature also suggests that the mechanism of positive interactions (e.g., habitat modification) and traits of the organisms that are facilitating or being facilitated may be equally important as environmental context in explaining positive interaction strength across space and time (Albertson & Allen, 2015; Cameron, Coulson, & Marshall, 2019; Hastings et al., 2007; Lam & Chisholm, 2020; Schöb, Armas, Guler, Prieto, & Pugnaire, 2013; Subalusky & Post, 2019).

Integrating the mechanism of positive interaction and traits of organisms that are positively interacting into environmental contexts such as the SGH has reinforced our ability to measure and predict the strength of positive interactions (Irving & Bertness, 2009; Miriti, 2006). Organismal activity, especially ecosystem engineering (Table 1) that leads to habitat and resource modification, can result in strong positive interactions (Jones, Lawton, & Shachak, 1994; Romero, Gonçalves‐Souza, Vieira, & Koricheva, 2015). Importantly, different mechanisms of positive interaction conferred through ecosystem engineering, such as habitat modification or resource modification, can operate at distinct and predictable spatio‐temporal extents across taxa and ecosystems (Atkinson, Allen, Davis, & Nickerson, 2018; Hastings et al., 2007). Additionally, variation in traits of both facilitators and beneficiaries can change the strength of positive interactions over space and time (Albertson & Allen, 2015; Cameron et al., 2019; Irving & Bertness, 2009; Moore, 2006; Schöb et al., 2013). Despite the progress summarized here, there is still need for a formal attempt to incorporate how these complex, yet predictable, spatio‐temporal dynamics may regulate the importance of positive interactions across a wide range of organisms and ecosystems. Here, we show how consideration of (1) the specific mechanisms of positive interaction and (2) organism traits can be used to improve integration of spatio‐temporal complexity into our understanding of positive interactions.

### 2.1 Approach 1: Mechanistic extent of positive interaction effects in space and time

We have only begun to consider and understand how the effects of positive interactions may extend across space (e.g., millimeters to landscapes) and time (e.g., daily to annual; Booth, Hairston, & Flecker, 2020; Cornacchia et al., 2018) to facilitate organism performance and affect community‐level organization (e.g., facilitation cascades; Altieri, Silliman, & Bertness, 2007; Crotty & Angelini, 2020). Positive and negative organism interactions can structure populations and communities over broad spatio‐temporal extents (Altieri et al., 2007; Cornacchia et al., 2018; Creel, 2018; Crotty & Angelini, 2020). For example, concepts such as trophic cascades and landscapes of fear (Creel, 2018) have furthered our understanding of how consumptive and non‐consumptive negative interactions structure populations and communities across large spatio‐temporal scales. These same spatial and temporal considerations for positive interactions are important, though underdeveloped, and might be approached by considering the underlying mechanisms.

**FIGURE 1:**
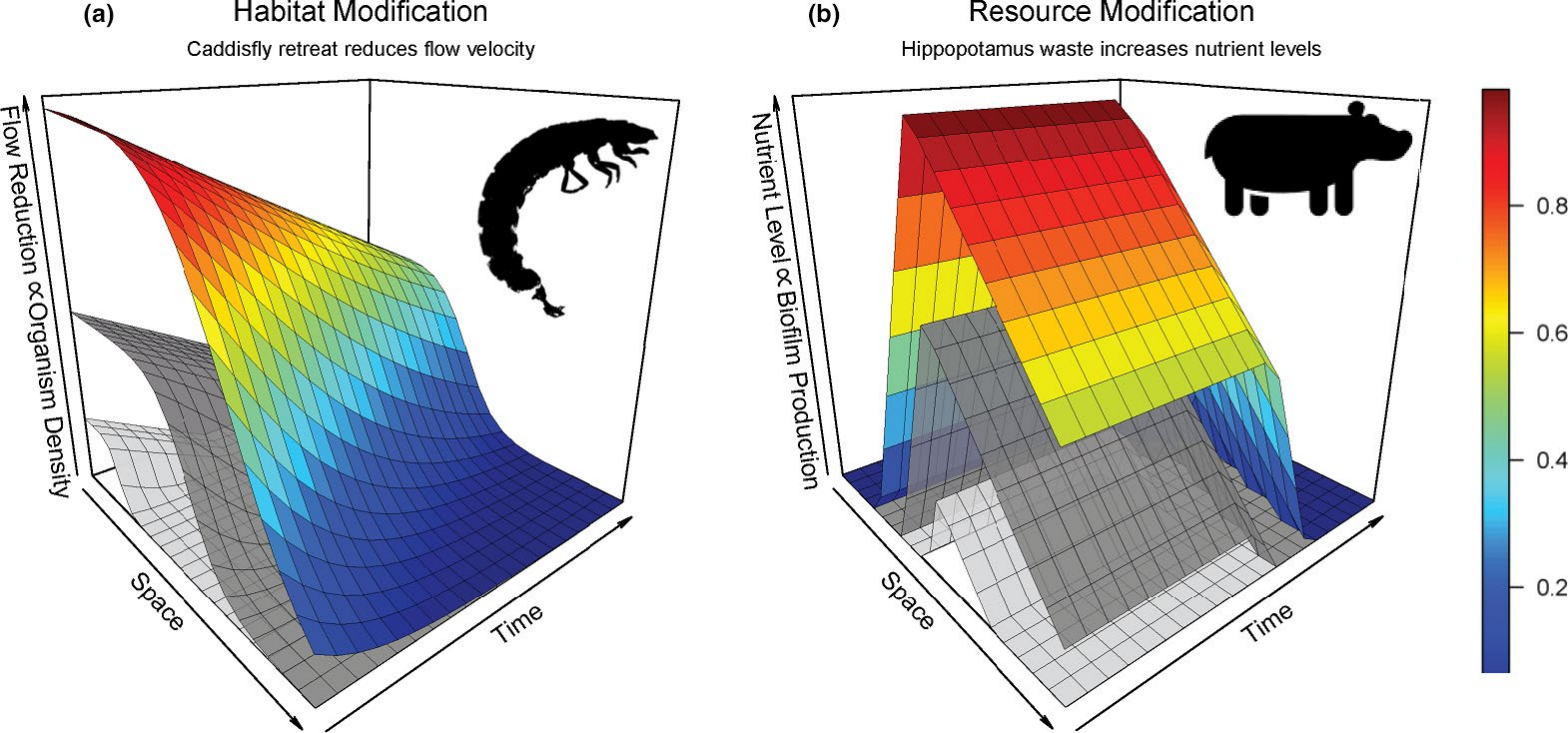
Spatio‐temporal response surfaces showing how traits of the facilitator modulate their effect on density of beneficiary organisms from two mechanisms of facilitation (a) habitat modification and (b) resource modification. The response is expressed on the *z*‐axis and also illustrated as a heat index represented from low (blue) to high (red) positive effect. The gray response surfaces under the heat index surface represent how trait variation of the facilitator may modify the magnitude of the overall positive effect through space and time. In this case, we are hypothesizing how the trait of facilitator body size may modulate the response surface. Color coding represents small (light gray), average (dark gray), and large (heat index) body size categories. (a) The numerical response (organism density) to habitat modification with data derived from the effects of caddisfly (Hydropsychidae) silk retreat structures on streambed hydraulics (Maguire et al., 2020). The response in invertebrate density is assumed to be proportional to the effect of the habitat modification on hydraulics and therefore greatest at the source and is maintained in space until, at some distance, the response declines quickly. The habitat modification is predicted to have slower temporal decay compared to resource modification based on maintenance of the retreat structure by the facilitator and robustness of the habitat modification to environmental degradation. The magnitude of this response is expected to be modulated by traits (body size) of the facilitator (gray shading). The largest bodied caddisflies should build the largest structures that have greater effect on hydraulics compared to smaller caddisflies. (b) The response of biofilm production to resource modification derived from data on subsides associated with hippopotamus urine and dung in rivers (Subalusky et al., 2018). The response peaks at some distance from the origin because river flow transports the nutrients downstream and declines rapidly thereafter. The decay over time is rapid once uptake begins because of high consumption rates. As in (a), this response surface is predicted to be modulated by traits (body size) of the facilitator, where larger bodied facilitators are predicted to produce more waste and have a greater positive effect

We hypothesize that the extent of positive interaction from the facilitator to beneficiaries will scale spatio‐temporally based on the mechanism of stress amelioration (Table 1; Figure 1a,b). The primary mechanisms of facilitation that reduce stress on the beneficiary are (a) physical habitat modifications that ameliorate biophysical stress (e.g., desiccation, temperature extremes) or provide predator refuge, and (b) resource modification by which organisms increase availability of resources (Jones et al., 1994; Lam & Chisholm, 2020). Improved description and prediction of positive interaction extent may lead researchers to better design quantitative metrics to characterize positive interactions when they result from these distinct mechanisms (Figure 1a,b).

Habitat modifications by ecosystem engineers are often fixed in space; however, the positive effects conferred by these modifications (e.g., amelioration of environmental stress) can extend further than the bounds of the structure itself, and persist temporally beyond the life of the engineer (Hastings et al., 2007; Jones et al., 1994). These habitat modifications can result from physical effects from the presence of an organism itself (e.g., trees, mussels, and corals that block wind or reduce water currents and wave action) or from an organism actively building or modifying aspects of the physical environment through the construction of burrows, mounds, or dams (Hastings et al., 2007; Jones et al., 1994; Silknetter et al., 2020). We predict that the amelioration of environmental stresses through habitat modifications will have a predictable spatial extent in terms of magnitude and direction (Figure 1a). Additionally, many environmental stressors, including wind, heat, high water velocity, or sediment debris flows, are directional and advective (Callaway et al., 2002; Cornacchia et al., 2018). Therefore, a stressed organism's orientation and proximity to an advective environmental stress (along with the direction and magnitude of the stress) relative to a facilitator may control the interaction outcome. For example, the distance “down wind” (0.5 m versus 2 m) of a wind‐shading plant makes the difference between a positive or competitive interaction (Trautz, Illangasekare, & Rodriguez‐Iturbe, 2017). Although we predict that physical habitat modifications have discrete and limited spatial extents because of their sessile nature, physical habitat modifications can have relatively large or extended temporal effects and occur over similar or longer time scales than negative biotic interactions (Figure 1a; Hastings et al., 2007; Tumolo, Albertson, Cross, Daniels, & Sklar, 2019). For example, negative behavioral territoriality or resource competition (e.g., interference) and trophic effects (e.g., predation) may last a matter of seconds (though these effects can propagate through generations), while a habitat modification structure left behind by an organism may persist and has direct positive effects several generations beyond the life of the ecosystem engineer (Hastings et al., 2007).

Resource limitation is a common ecological stressor and understanding the spatio‐temporal extent of benefit from organism‐mediated resource modification is an important source of variation to consider in delineating positive interactions (Figure 1b; Lam & Chisholm, 2020; Tilman, 1985). Organism‐mediated resource modification can occur through modification of resource state, form, and transport (Jones et al., 1994; Lam & Chisholm, 2020; Tilman, 1985). For example, bioturbation through the stirring up of sediments can increase availability and transport of food particles and nutrients otherwise unavailable to consumers (Moore, 2006; Silknetter et al., 2020). Additionally, fecal matter and its transport can be a significant source of carbon and nutrients for resource‐limited consumers such as microbial communities (Subalusky & Post, 2019). The spatial scope of resource modification can vary widely from micro‐habitat scale (e.g., <1 cm in caddisfly gardening; Lamberti, 1996) to landscape or ecosystem scale (e.g., 75 km in subsides from hippopotamus waste in rivers; Subalusky, Dutton, Njoroge, Rosi, & Post, 2018). The spatio‐temporal extent of resource modification may also be modulated by the relevant advective forces in the study system, such as ocean currents and river flows, along with variable consumer uptake rates as materials move through an environment (Figure 1b; Subalusky & Post, 2019).

In general, we predict a delayed decay in the spatial extent for resource‐mediated stressors compared to habitat modifications on a per‐capita basis (Figure 1a,b). The temporal extent of modified resources will be based partly on the demand and partly on the composition of organisms within resource‐limited communities (Subalusky & Post, 2019). We predict a more rapidly decaying temporal extent of positive effect from resource modification compared to habitat modification as food‐limited consumer populations and communities may rapidly consume these subsides (Figure 1b). These general, albeit simple, predictions provide a foundation from which positive interactions resulting from an individual habitat or resource modification will scale in space and time. Importantly, response surfaces (Figure 1) could be built upon from the individual level to incorporate more realistic population and community‐level scenarios. For example, multiple response surfaces could be created for the study of positive interaction at the population and community levels, where numerous or simultaneous habitat and resource modifications from multiple or the same species could interact on the landscape (Hastings et al., 2007; Sanders et al., 2014). Additionally, response surfaces could incorporate fluctuating environmental conditions, along with community assembly history to better understand the effects of ecological hysteresis (HilleRisLambers et al., 2013). Furthermore, it is well established that both positive and negative biotic effects can occur through the modification habitats and resources (Hastings et al., 2007; Jones et al., 1994) and the response surface could be leveraged to investigate these simultaneously occurring negative or neutral effects of modifications, and feedbacks to population dynamics of facilitators and beneficiaries alike (Cameron et al., 2019; Tilman, 1985).

### 2.2 Approach 2: Organism traits in space and time

Understanding organism traits and sources of trait variation can be used to more specifically identify how members of populations and communities will respond to stress, confer positive effects, or benefit from a particular positive interaction across space and time (Figure 1a,b). The effect of a stressor is always relative to individuals that can be grouped together based on similar traits (Mouillot, Graham, Villéger, Mason, & Bellwood, 2013; Violle et al., 2007). Additionally, traits of facilitators will control the type, size, and durability of habitat modification or resource modification and ultimately its positive effects on recipient communities (Figure 1a,b; Albertson & Allen, 2015; Cameron et al., 2019). Here, we discuss how a trait‐based approach can be combined with the mechanistic reach of benefit response surface to further understand the spatio‐temporal effect of positive interactions for a variety of taxa expressing both intra‐and‐interspecific trait variation.

Organism traits and stressors are not constant over space and time (Maestre et al., 2009; McGill, Enquist, Weiher, & Westoby, 2006; Romero, 2004); therefore, incorporating spatio‐temporal trait variation of facilitators and beneficiaries into our understanding of stress amelioration by positive interactions will be an important step forward. For example, facilitator and beneficiary traits vary across space due to environmental heterogeneity in biophysical stress and resource availability (Anderegg, 2015; Trautz et al., 2017). Additionally, facilitator and beneficiary traits can express temporal variation through ontogeny and age‐specific size structuring (Figure 1a,b; Callaway & Walker, 1997; Doxford, Ooi, & Freckleton, 2013; Miriti, 2006; Tewksbury & Lloyd, 2001). These sources of spatio‐temporal trait variation will act to contextualize the degree to which facilitators modify habitat and resources along with the ability of beneficiaries to utilize such modifications (Figure 1a,b). For example, facilitator body size is one such trait that will determine the dimensions of a habitat modification (Kinlaw & Grasmueck, 2012), while the body size of a beneficiary will determine their ability to utilize such a habitat modification and seek refuge from environmental stressors. Therefore, facilitator traits and beneficiary traits together should determine the overall strength of positive interaction from a habitat or resource modification. Importantly, body size of facilitators and beneficiaries may express intra‐and interspecific temporal variation through life history stage and phenology, respectively as well as spatial variation across gradients of environmental conditions, resource availability, and community composition. Inclusion of organism traits and respective sources of variation into positive interaction frameworks offers an exciting opportunity to better understand positive interactions over an organism's lifetime and across landscapes (McGill et al., 2006; Mouillot et al., 2013; Violle et al., 2007).

## 3 SPATIO‐TEMPORAL RESPONSE SURFACES OF HABITAT AND RESOURCE MODIFICATIONS

A response surface is a mathematical technique that is used to measure the effect of multiple factors operating simultaneously on a response of interest (Albert et al., 2010). Here, we leverage the response surface to visualize the three‐dimensional positive interaction response over both spatial and temporal axes for habitat and resource modification. The examples of response surfaces that we present were derived from field and laboratory measurements and they help demonstrate how researchers could translate spatial and temporal data into a response surface for analysis. Importantly, the shape of these response surfaces would differ depending on the study system (Albert et al., 2010).That is, the linear decay across the time axis represented in our response surfaces might be expressed as an exponential decay in another system, and the functional form of the decay across the spatial extent might also differ with environmental context or for organisms with different traits.

The habitat modification example (Figure 1a) is based on published spatio‐temporal effects of caddisfly retreats on boundary‐layer hydraulics (Friedrichs & Graf, 2009; Maguire, Tumolo, & Albertson, 2020). The caddisfly retreats themselves are centimeter‐scale structures composed of organic and inorganic material held together by silk constructed for shelter and passive filter feeding within river ecosystems. Retreats are built upon benthic substrates where they confer positive effects to other stream insects by providing local refugia from higher surface flow velocities over space and over time (Figure 1a; Maguire et al., 2020; Nakano, Yamamoto, & Okino, 2005). In this example, we make the assumption that the effect of the structure on the physical environment is proportional to its positive biotic effect in terms of a density response of beneficiaries (Figure 1a). Spatially, the caddisfly retreat (or any habitat modification with similar physical properties) causes a reduction in flow velocity immediately downstream of the structure by protruding into the water column and inducing local current blocking and wake formation (Davis & Barmuta, 1989; Friedrichs & Graf, 2009; Maguire et al., 2020) until the flow reduction diminishes at a certain distance downstream from the structure (Friedrichs & Graf, 2009). This area of lower flow velocity is taken advantage of by stream invertebrates (Nakano et al., 2005). The temporal dimension of the positive effect is expected to be upheld through maintenance by the caddisfly and following abandonment of the structure where the retreat continues to provide positive benefit for other stream invertebrates (Tumolo et al., 2019). Upon abandonment of the retreat due to drift or death of the caddisfly, the retreat structure degrades, starts to reduce in size, and thus its effect on flow reduction diminishes correspondingly (Maguire et al., 2020). The structural decay occurs linearly over time (from 14 mm to 8 mm) but is still physically present over 60 days following abandonment (Maguire et al., 2020). In this caddisfly retreat example (Figure 1a), the time axis on the response surface has a low rate of decay and a “long tail,” both of which are statistically measurable properties of the response surface that could then be compared to properties of response surfaces derived across a range of intra‐ and interspecific traits.

The resource modification response surface is based on a known relationship between hippopotamus waste (urine and dung) and increasing ammonium (NH4+) availability (a limiting nutrient) flowing down river, imparting a positive effect on gross primary production of biofilm (Figure 1b; Subalusky et al., 2018). Hippopotamus urine and dung have been shown to increase NH4 + availability for 71 hr, before complete uptake in 133 hr based on evidence from chamber experiments (see Figure 1a in Subalusky et al., 2018). In addition, hippo urine and dung increase NH4+ availability downstream of the source (up to ~75 river km, with a peak at 20 km downstream) via advective forcing from river flow (Figure 4a in Subalusky et al., 2018). We combine this evidence to develop the response surface for the effect of urine and dung‐NH4 + on the simulated positive response by biofilm production.

For both of these examples, our foundational hypothesis is that the spatio‐temporal extent of positive interactions depends on whether the underlying mechanism is habitat or resource modification. We can argue, for example, that the relative rate of decline in the response surface over the time axis is quicker for resource augmentation than it is for habitat modification. Under the assumptions for the example response surfaces described above, the relative rate of linear decline along the time axis is −2 per unit time for the NH4+ from hippopotamus waste, whereas its only −0.025 per unit time for flow refuge from structural decay of the caddisfly retreat. Although these representative examples from field and laboratory studies are just two of many, they accurately represent how different mechanisms of positive interaction can have orders of magnitude differences in the temporal rates of decay in the response surfaces. Nevertheless, our hypothesis leads to a number of predictions that can be tested to investigate how these two mechanisms of positive interactions will differentially operate in nature. We predict that response surfaces may differ greatly among the (a) response metrics being studied (density, biomass, growth rate, species diversity), (b) mechanism of facilitation operating (habitat modification, resource modification; Figure 1a,b), and (c) traits of organisms facilitating or being facilitated (Figure 1a,b). The response surfaces are a mathematical and visualization technique from which characteristics such as slope and magnitude of the surface can be extracted and analyzed. From our perspective, this technique provides a clear way to integrate data into better understanding of positive interactions over space and time.

## 4 INTEGRATING APPROACHES THROUGH MEASUREMENT AND ANALYSIS

**FIGURE 2:**
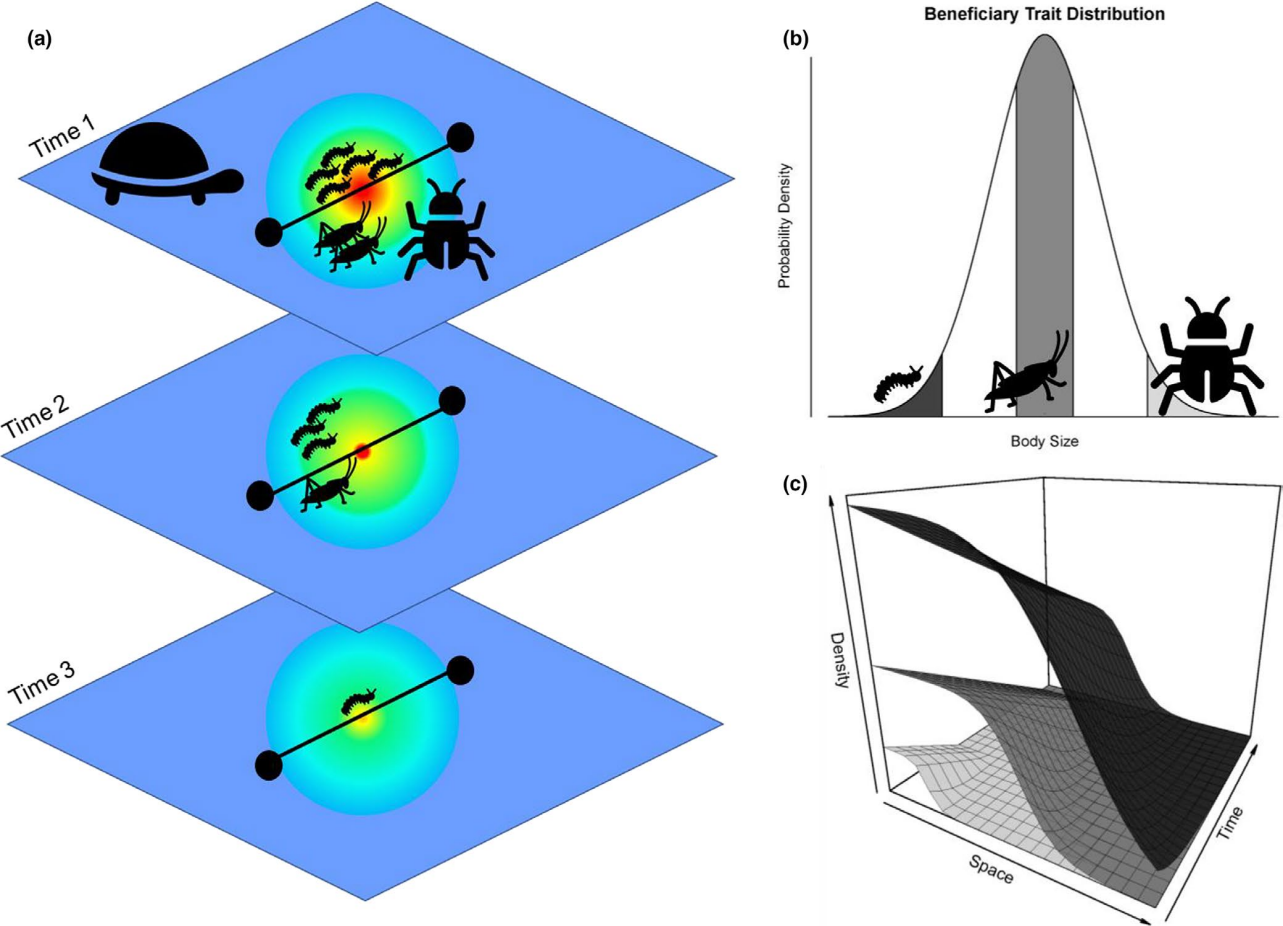
Survey techniques to collect data on the spatio‐temporal extent of positive interactions from a habitat modification example that can be used to create response surfaces and test the predictions of the mechanistic and trait‐based hypotheses. (a) A turtle digs a burrow (habitat modification) which confers positive effect on a suite of insect species by providing a thermal refuge from high heat and forest fires (Kinlaw & Grasmueck, 2012). Transects (black lines) can be used to measure physical parameters of this habitat modification over space and over a time series. Along these transects, researchers can measure the positive response of organisms and directly inform the spatio‐temporal axes of a response surface. Based on this example, the positive effect of the structure decays spatially with distance away from the center of the burrow. Over time the spatial extent of the positive effect is further constrained as the burrow is reduced in size through weathering. Trait measurements, in this case body size, are recorded for organisms comprising the density response (beneficiaries) along a transect of positive interaction extent. (b) beneficiaries can be categorized based on a chosen trait, such as body size (dark gray = small, gray = medium, light gray = large). (c) The numerical or functional response to positive interactions for organisms that vary in a specified trait value can be expressed as a response surface. In this example, insect beneficiaries with the smallest body sizes experience the greatest spatio‐temporal effects from the habitat modification

The response surfaces provide the framework from which to evaluate the magnitude and shape of positive effects over space, time, among individuals varying by trait (Figure 1), and across environmental conditions (e.g., SGH). However, before any analyses can be conducted, the response surfaces must be derived from data. The studies used in our examples (Figure 1) were effective for demonstrating that these effects occur over certain space–time dimensions in nature; however, these examples were not designed to build testable response surfaces. In the interest of motivating the development of more complete and testable response surfaces, we outline how field measurement data can be collected by researchers to develop such spatio‐temporal response surfaces in future work (Figure 2).

The spatial and temporal extents of habitat and resource modifications and their positive effects on communities can be measured using traditional survey techniques with any spatially explicit array such as transects and quadrats in natural ecosystems along with field and laboratory experiments (Figure 2a). Survey techniques can be used to sample for response variables such as functional responses of rates of growth or numerical responses of density and biomass to fit an empirically derived response surface for a single, or multiple, species varying along a trait axis, such as body size (Figure 2a–c). The transect data provide the evidence of the response surface over the spatial dimension, and if repeated, transects can provide the evidence for the spatial patterns over time (Figure 2a). For each transect, an additional set of measurements would record the physical dimensions of a structure or resource (Figure 2a), providing the necessary data to determine whether the magnitudes or changes in physical dimensions of a mechanism of facilitation (habitat and resource modification) affect the response surface in space or time. For example, if one was measuring the effect of habitat modification such as density of insects using a burrow, then measurements of an animal's burrow size could be recorded during each transect as a covariate to the response (Figure 2a, Kinlaw & Grasmueck, 2012). In the case of resource modification, organisms that are modifying the availability and transport of resources could be fed isotopically labeled food (Allen, Vaughn, Kelly, Cooper, & Engel, 2012; Atkinson, Kelly, & Vaughn, 2014). Then, samples of the response variables should show evidence of assimilated isotopes if beneficiaries are consuming and provisioning the resource to biomass (Allen et al., 2012; Atkinson et al., 2014). As such, the isotopic signature can be tracked via transect samples to derive the response surface of isotopic assimilation, as a proxy for the functional response of consumption and growth, to establish the spatial and temporal extent of influence of a resource (Allen et al., 2012; Atkinson et al., 2014). Although isotopes would only indicate that the resource has been assimilated and not necessarily that it has enhanced performance, efforts could be made to track labeled individuals or populations and quantify their growth or reproductive success. Considering the spatio‐temporal extent of positive interactions in these ways allows for a positive effect, based on the response surface, to be linked to physical dimensions of a particular habitat modification or magnitude of a resource modification.

## 5 APPLICATION AND RESTORATION: INTEGRATING CONCEPTS INTO THE FIELD AND LAB

One of the greatest challenges facing ecologists is the prevention of and response to rapid biodiversity loss and the subsequent deterioration of ecological interaction networks. Harnessing the power of existing positive interaction relationships can improve how we manage, restore, and conserve organisms and ecosystems (Halpern, Silliman, Olden, Bruno, & Bertness, 2007; Rodriguez, 2006). For example, changing management strategies to embrace positive interaction instead of minimizing competition has resulted in markedly higher (50% increase in replanting growth) restoration success of coastal ecosystems (Derksen‐Hooijberg et al., 2018; Silliman et al., 2015). Consideration of the spatio‐temporal dependence of positive interactions will also have important implications for the timing, placement and ultimately the efficacy of management strategies (Calatayud et al., 2019; Fischman, Crotty, & Angelini, 2019). The window of effective implementation of restoration techniques that include positive interactions is likely spatio‐temporally constrained, especially in successional‐based restoration efforts related to trait‐based ontogenetic shifts in organism relationships (Figures 1 and 2). Evidence for such a relationship is already resulting from re‐vegetation efforts (e.g., grasslands, forests and marshes; Bersoza Hernández et al., 2018; Derksen‐Hooijberg et al., 2018; Padilla & Pugnaire, 2006; Silliman et al., 2015) and coastal shoreline restoration projects (Bersoza Hernández et al., 2018; Fischman et al., 2019). However, more work is needed to identify which types of ecosystems or organisms might show the largest successes from restoration strategies that include explicit spatio‐temporal positive interactions. Management strategies may also benefit from considering a mechanistic spatio‐temporal extent. For example, the proximity of seedlings during re‐vegetation efforts could alter the strength of positive interactions (Derksen‐Hooijberg et al., 2018; Fischman et al., 2019; Trautz et al., 2017). In resource‐limited populations, management of resource flows and sources is important. For example, targeting an increase in habitat connectivity for facilitators may be an important direction forward if such facilitators are known to impart a strong positive interaction by transporting resources around the landscape (Figure 1b; Subalusky & Post, 2019). Additionally, traits of positively interacting organisms (e.g., size structure) that are plastic in response to environmental change could be explicitly considered in conservation strategies for rare or keystone species. For example, several positive interaction networks are ontogenetically structured (Callaway & Walker, 1997; Miriti, 2006); therefore, such relationships could be lost or altered by climate‐driven phenological mismatch. Such trait‐based considerations may provide adaptive, testable hypotheses for better understanding relationships and shifts in relationships between facilitators and beneficiaries, while also offering an opportunity to implement restoration strategies that are robust to predicted future environmental changes (Callaway & Walker, 1997). Furthermore, investigations interested in positive interactions over greater spatio‐temporal scales, including long‐lived organisms or large distance migrations, could benefit by leveraging long‐term datasets that include spatially broad and diverse sampling locations. The field of applied positive interactions is too rich with opportunity to exhaustively cover here; however, we hope that the two approaches we highlight (mechanistic extent and trait based) provide an accessible and pragmatic direction forward to integrating spatio‐temporal dynamics of positive interactions into management and conservation.

## 6 CONCLUSIONS AND FUTURE DIRECTIONS

Future directions for positive interactions in ecology include exciting research questions and opportunities for advancement. We have largely focused this paper on macro‐organisms and suggest that future work might test whether our predictions hold for micro‐organisms in positive interaction networks. For example, microbes are known to substantially alter physical habitat through bioerosion and cohesion and alter nutrient availability (Atkinson et al., 2018), but it is unknown whether there would be similar differences in space–time response surfaces at the micro‐scale between different mechanisms of facilitation. An additional important step forward will be to explicitly consider interactions between habitat modification and resource modification mechanisms because physical habitat modifications often change availability of resources and trophic interactions (Kéfi et al., 2012; Sanders et al., 2014). Exciting landscape questions also exist, such as how the dispersal and mobility of positively interacting organisms modulates spatio‐temporal extent and magnitude of benefit. Movement of ecosystem engineers can greatly expand the spatio‐temporal scope of influence associated with structures because a single individual can create multiple habitat modifications over large spatial distances that can persist on the landscape for long temporal scales (Booth et al., 2020); what would be the collective effect of these engineers on the landscape in both space and time? Furthermore, how do spatial configurations (e.g., corridor versus dendritic) of landscapes interact with environmental and biological stressors to regulate landscape level interactions? Lastly, the study of eco‐evolutionary dynamics could be especially pertinent in understanding the role of intra‐and interspecific trait variation within positive interaction networks and the consequence of such trait variation at the individual, population, community, and ecosystem levels. This paper synthesizes current knowledge and provides novel, explicit approaches for integrating spatio‐temporal dynamics into positive interaction frameworks. Spatio‐temporal approaches in ecology are becoming increasingly important and deserve further development during this time of unprecedented global change.

## CONFLICT OF INTEREST

The authors declare they have no conflicts of interest regarding this work.

## AUTHOR CONTRIBUTION

**Benjamin B. Tumolo:** Conceptualization (lead); Visualization (lead); Writing‐original draft (lead); Writing‐review & editing (lead). **Leonardo Calle:** Conceptualization (lead); Visualization (lead); Writing‐original draft (supporting); Writing‐review & editing (supporting). **Heidi Anderson:** Conceptualization (equal); Visualization (supporting); Writing‐original draft (supporting); Writing‐review & editing (supporting). **Michelle Briggs:** Conceptualization (equal); Visualization (supporting); Writing‐original draft (supporting); Writing‐review & editing (supporting). **Sam Carlson:** Conceptualization (equal); Visualization (supporting); Writing‐original draft (supporting); Writing‐review & editing (supporting). **Michael MacDonald:** Conceptualization (equal); Visualization (supporting); Writing‐original draft (supporting); Writing‐review & editing (supporting). **J. Holden Reinert:** Conceptualization (equal); Visualization (supporting); Writing‐original draft (supporting); Writing‐review & editing (supporting). **Lindsey K. Albertson:** Conceptualization (lead); Funding acquisition (lead); Visualization (lead); Writing‐original draft (lead); Writing‐review & editing (lead).

### OPEN RESEARCH BADGES

This article has been awarded Open Data, Open Materials, Preregistered Research Designs Badges. All materials and data are publicly accessible via the Open Science Framework.

## Data Availability

All data used were accessed through referenced literature.
